# Luftverschmutzung und Herz-Kreislauf-Erkrankungen

**DOI:** 10.1007/s00059-020-05016-9

**Published:** 2021-01-18

**Authors:** Thomas Münzel, Omar Hahad, Andreas Daiber, Jos Lelieveld

**Affiliations:** 1 Zentrum für Kardiologie – Kardiologie I, Universitätsmedizin der Johannes-Gutenberg-Universität Mainz, Langenbeckstraße 1, 55131 Mainz, Deutschland; 2 Max-Planck-Institut für Chemie, Johannes-Gutenberg-Universität Mainz, Mainz, Deutschland

**Keywords:** Luftschadstoffe, Feinstaub, Kardiovaskuläres Risiko, Vorzeitige Mortalität, Prävention, Air pollutants, Particulate matter, Cardiovascular risk, Premature mortality, Prevention

## Abstract

Die Luftverschmutzung in der Umgebung und in Haushalten ist weltweit jährlich für mittlerweile knapp 9 Mio. vermeidbare, vorzeitige Todesfälle und innerhalb Europas für knapp 800.000 solcher Todesfälle verantwortlich. Die Luftverschmutzung verkürzt somit weltweit die Lebenserwartung um knapp 3 Jahre. Das Rauchen, ein nachgewiesener Herz-Kreislauf-Risiko-Faktor, verkürzt die mittlere Lebenserwartung um 2,2 Jahre. Epidemiologische Studien zeigen, dass Luftverschmutzung durch Feinstaub mit erhöhter kardiovaskulärer Morbidität und Mortalität assoziiert ist. Hierfür verantwortlich sind hauptsächlich durch Feinstaub ausgelöste oder verschlimmerte Herz-Kreislauf-Erkrankungen, wie koronare Herzkrankheit (KHK), Herzinfarkt, Herzinsuffizienz, Schlaganfall, Hypertonie und auch Diabetes. Feinstaubpartikel können nach Inhalation zum einen direkt ins Gehirn und zum anderen über einen Transitionsprozess in die Blutbahn gelangen. Dort werden sie in die Blutgefäße aufgenommen, wo sie die Bildung reaktiver Sauerstoffspezies (ROS) in der Gefäßwand stimulieren. Damit begünstigen sie die Entstehung atherosklerotischer Veränderungen und steigern so das kardiovaskuläre Risiko, insbesondere eine Zunahme an chronisch-ischämischer Herzerkrankung und Schlaganfall. Neuere Untersuchungen berichten, dass bei COVID-19(„coronavirus disease 2019“)-Patienten ein hoher Grad an Luftverschmutzung mit schweren Krankheitsverläufen mit kardiovaskulären Komplikationen und Lungenerkrankungen korreliert. Dies macht präventive Maßnahmen, wie z. B. eine Absenkung der Grenzwerte für Luftschadstoffe, erforderlich. Individuelle Maßnahmen, um die gesundheitlichen Folgen von Feinstaub abzumildern, werden ebenfalls diskutiert.

## Abstract (en)

Air pollution in the environment and in households is responsible worldwide for almost 9 million preventable premature deaths per year and almost 800,000 such deaths within Europe. Air pollution therefore shortens life expectancy worldwide by almost 3 years. Smoking, a proven cardiovascular risk factor, shortens the mean life expectancy by 2.2 years. Epidemiological studies have shown that air pollution from fine and coarse particulate matter is associated with increased cardiovascular morbidity and mortality. Responsible for this are mainly cardiovascular diseases, such as coronary heart disease, heart attack, heart failure, stroke, hypertension and also diabetes, which are mainly caused or aggravated by fine particulate matter. After inhalation fine particulate matter can reach the brain directly and also reach the bloodstream via a transition process. There, the particles are absorbed by the blood vessels where they stimulate the formation of reactive oxygen species (ROS) in the vascular wall. They therefore promote the formation of atherosclerotic changes and in this way increase the cardiovascular risks, especially an increase in chronic ischemic heart disease and stroke. Recent studies also reported that in coronavirus disease 2019 (COVID-19) patients a high degree of air pollution is correlated with severe disease courses with cardiovascular complications and pulmonary diseases. This necessitates preventive measures, such as lowering of the upper limits for air pollutants. Individual measures to mitigate the health consequences of fine particulate matter are also discussed.

Die Verschmutzung der Umgebungsluft (im Freien, „ambient air pollution“) und der Luft im Haushalt (Innenluft) wird als Hauptrisikofaktoren für vorzeitigen Tod und Morbidität mit erheblichen direkten und indirekten Kosten für die Gesellschaft angesehen [1]. Die Weltgesundheitsorganisation (World Health Organization [WHO]; [2]) bzw. das Projekt Global Burden of Disease (GBD; [1]) berechnete aufgrund von Luftverschmutzung mit Feinstaub der Kategorie PM_2,5_ („particulate matter“ mit Teilchengröße < 2,5 µm) 4,2 Mio. vermeidbare Todesfälle pro Jahr weltweit. Mehr als die Hälfte der Todesfälle war dabei die Folge von Herz-Kreislauf-Erkrankungen, wie z. B. ischämische Herzerkrankung oder Schlaganfall, bzw. von anderen nicht übertragbaren Krankheiten, wie Diabetes, Bluthochdruck, Lungenkrebs und chronisch-obstruktive Lungenerkrankung („chronic obstructive pulmonary disease“, COPD).

Nach neuen Erkenntnissen und der Einführung einer aktualisierten Hazard-Ratio(HR)-Funktion für PM_2,5_, auf Basis des sog. Global Exposure Mortality Model (GEMM), ergaben sich drastisch höhere Zahlen von 8,8 bis 8,9 Mio. vorzeitigen Todesfällen pro Jahr aufgrund der Verschmutzung der Umgebungsluft. Damit hat sich die berechnete Zahl von jährlichen vorzeitigen Todesfällen nahezu verdoppelt [3, 4].

Wir wollen mit unserem Übersichtsartikel zu den Auswirkungen von Luftverschmutzung auf das Herz-Kreislauf-System den Leser auf den neuesten Stand bringen sowie die akuten (kurzfristigen) und chronischen (langfristigen) Belastungen durch Luftverschmutzung erörtern. Auch werden aktuelle Daten diskutiert, die darauf hindeuten, dass die weltweite Mortalität und Morbidität, bedingt durch Herz-Kreislauf-Erkrankungen und andere nicht übertragbare Krankheiten, aufgrund von PM_2,5_-Verschmutzung noch viel größer ist, als zuvor von den beiden wichtigsten Informationsquellen, d. h. WHO und GBD, berechnet wurde.

## Was verstehen wir unter Luftverschmutzung?

Unter „ambient air pollution“ wird eine komplexe Mischung einer Vielzahl von Komponenten subsumiert. Dazu gehören PM sowie gasförmige Schadstoffe wie Ozon (O_3_), Stickstoffdioxid (NO_2_), flüchtige organische Verbindungen (inkl. Benzol), Kohlenmonoxid (CO) und Schwefeldioxid (SO_2_; [5, 6]).

Primärverunreinigungen wie Rußpartikel sowie Stickstoff- und Schwefeloxide werden durch Verbrennung fossiler Brennstoffe direkt in die Luft abgegeben. Hauptquellen für NO_2_ sind der motorisierte Straßenverkehr, die Stromerzeugung, Industriequellen und die Beheizung von Wohnräumen. Sekundäre Schadstoffe werden in der Atmosphäre von anderen Komponenten gebildet, in der Regel chemisch aus Gasen, die direkt emittiert werden und daher als Primärschadstoffe gelten, sowie durch hochenergetische Ultraviolett(UV)-Strahlung, die in der Atmosphäre eine chemische Toxifizierung der Partikeloberflächen und von flüchtigen organischen Substanzen verursacht (z. B. zu zahlreichen krebserregenden Endprodukten; [6–8]).

**Figure 1:**
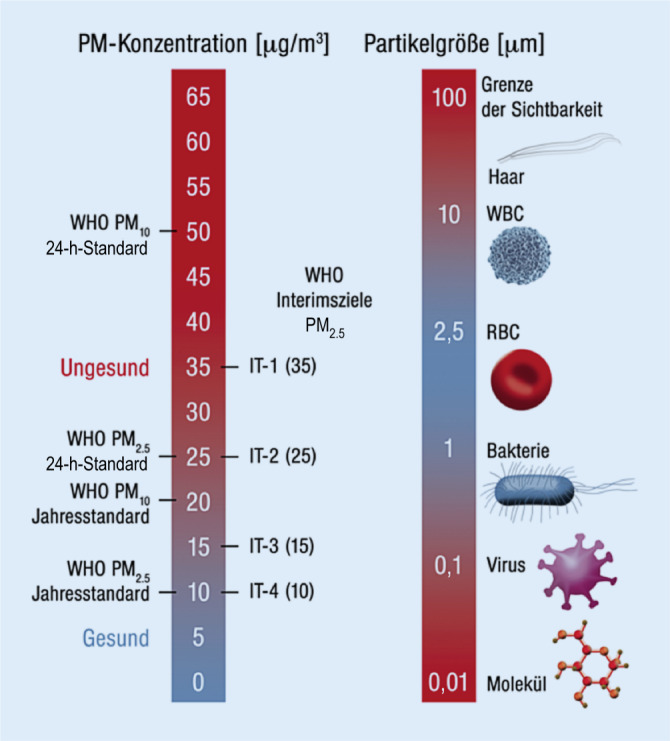

Ein wichtiges Beispiel ist Ozon, das durch komplexe photochemische Reaktionen von Stickoxiden und flüchtigen organischen Komponenten gebildet wird. Feinstaub besteht teilweise aus Primärpartikeln, aber insbesondere die kleinsten Teilchen werden hauptsächlich durch chemische Reaktionen in der Atmosphäre gebildet/modifiziert und sind als Sekundärpartikel zu bezeichnen. Die Vorläufergase und -partikel werden aus einer Vielzahl von Quellen freigesetzt. Feinstaub wird in 3 Hauptgrößenklassen eingeteilt (Abb. 1; [6–8]):

- relativ große Partikel (Durchmesser: ≤ 10 und ≥ 2,5 µm; PM_10_), die damit die Größe einer Zelle besitzen;
- feine Partikel (Durchmesser: ≤ 2,5 und ≥ 0,1 µm; PM_2,5_), entsprechend der Größe eines roten Blutkörperchens;
- ultrafeine Partikel (Durchmesser: < 0,1 µm; PM_0,1_), der Größe eines Virus entsprechend.

Wichtige Quellen für Primärpartikel, die typischerweise aus einem Kohlenstoffkern bestehen, sind der motorisierter Straßenverkehr, Kraftwerke, Heizgeräte für Industrie und Privathaushalte, die Öl, Kohle oder Holz verwenden, sowie insbesondere in Deutschland die Landwirtschaft, wo v. a. Überdüngung zu hohen Feinstaubkonzentrationen führt. Die Verbrennungsprozesse führen zu PM_2,5_, der aus elementarem Kohlenstoff, Übergangsmetallen, Endotoxinen aus Bakterien oder Pilzen, komplexen organischen Molekülen, Sulfat, Nitrat und Ammonium besteht, wobei letztere Komponenten in der Atmosphäre aus flüchtigen organischen Verbindungen, SO_2_, NO_2_ und Ammoniak (NH_3_) gebildet werden [7].

Feinstaubteilchen können große Entfernungen (bis zu mehreren 100 km) zurücklegen, was zu hohen Hintergrundkonzentrationen in einem weiten Bereich führen kann. So berechneten Lelieveld und Mitarbeiter, dass natürliche Feinstaubquellen relativ stark (18 %) zur Sterblichkeit aufgrund der Luftverschmutzung beitragen (ca. 750.000 pro Jahr), die zu einem großen Teil durch Wüstenstaub in der Luft verursacht wird [9]. Allerdings spielen die Wüstenstaubpartikel als PM_2,5_ in Deutschland eine untergeordnete Rolle.

Die zeitliche Variation der täglichen durchschnittlichen Luftverschmutzungskonzentrationen hängt hauptsächlich mit den Witterungsbedingungen zusammen, die die Verteilung der Verschmutzung beeinflussen, und weniger mit Schwankungen der Intensität der Schadstoffquellen. Wichtige Faktoren sind Windrichtung, Windgeschwindigkeit und atmosphärische Stabilität. Die Luftverschmutzungskonzentrationen variieren ebenfalls innerhalb eines Tages, z. B. infolge des Wachstums der Mischungsschicht durch die Sonneneinstrahlung. Es wird auch vermutet, dass die Feinstaubteilchen die durch Atmen und Sprechen (und Singen) erzeugt werden, als Vektor für das Verteilen des Coronavirus SARS-CoV‑2 („severe acute respiratory syndrome coronavirus 2“) verantwortlich sein können (siehe Abschnitt „COVID-19 und Luftverschmutzung“).

## Auswirkungen von Luftverschmutzung auf das Herz-Kreislauf-System

Bei der Diskussion der pathophysiologischen Auswirkungen von Luftverschmutzung wird im Folgenden auf PM_2,5_ fokussiert, weil v. a. die kleinen Feinstaubteilchen tief in die Lunge vordringen können. Nach Inhalation von PM_2,5_ wird der Feinstaub über einen Transitionsprozess von den Alveolen durch die Membran der Lungenepithelzellen in die Blutbahn und anschließend in die Gefäßwände aufgenommen [7]. Der Feinstaub stimuliert in der Gefäßwand die Bildung reaktiver Sauerstoffspezies („reactive oxygen species“, ROS), also von prooxidativen Substanzen und proinflammatorischen biologischen Mediatoren (d. h. solche Zytokine wie Interleukin[IL]-6 und Tumornekrosefaktor [TNF]) und Akute-Phase-Proteinen wie C‑reaktives Protein (CRP) und dem Vasokonstriktorhormon Endothelin‑1 [7]. Diese Prozesse initiieren bzw. beschleunigen atherosklerotische Veränderungen. Man geht heute davon aus, dass die Wahrscheinlichkeit einer raschen Translokation von den Partikeln in die Gefäßwand desto größer ist, je kleiner (bis hin zu Ultrafeinstaub mit 0,1 µm) der Partikel ist [10]. Weiterhin wird eine Aktivierung der Blutgerinnung beobachtet. Dementsprechend sind hohe PM_2,5_-Konzentrationen von Hyperkoagulationsbiomarkern wie hohen Plasmaspiegeln von Fibrinogen und D‑Dimer sowie von einer verstärkten Thrombinbildung begleitet [11].

Humanexperimentelle Daten zeigen eine inverse Beziehung zwischen der PM-Exposition und der Variabilität der Herzfrequenz. PM_2,5_ kann über direkte sympathikusaktivierende Wirkungen im Gehirn [8] bzw. durch Auslösung einer endothelialen Dysfunktion aufgrund einer verminderten Bioverfügbarkeit vaskulären Stickstoffs (NO) eine arterielle Hypertonie auslösen [7].

## Sterblichkeit durch Luftverschmutzung deutlich höher als bisher angenommen?

Die beiden Hauptquellen für Daten zur globalen Mortalität und Morbidität durch Luftverschmutzung sind WHO und GBD, deren Schätzung für durch PM verursachte Todesfälle bei etwa 4,2 Mio. lag [1, 2]. Beide Quellen stimmten darin überein, dass Herz-Kreislauf-Erkrankungen die Hauptursache für PM_2,5_-induzierte Todesfälle darstellen. 2018 und 2019 berechneten 2 neue Studien eine viel höhere Mortalitäts- und Morbiditätsbelastung als bisher angenommen, insbesondere aufgrund der Verwendung des GEMM, einer neuen und umfassenderen HR-Funktion der PM_2,5_-Konzentrations-Gesundheitseffekt-Beziehung [3, 4]. Das GEMM zielt auf alle möglichen Krankheiten, die durch Luftverschmutzung verursacht oder verschlimmert werden, weil die WHO und GBD sich auf eine beschränkte Zahl, spezifischer Krankheiten beziehen.

**Figure 2:**
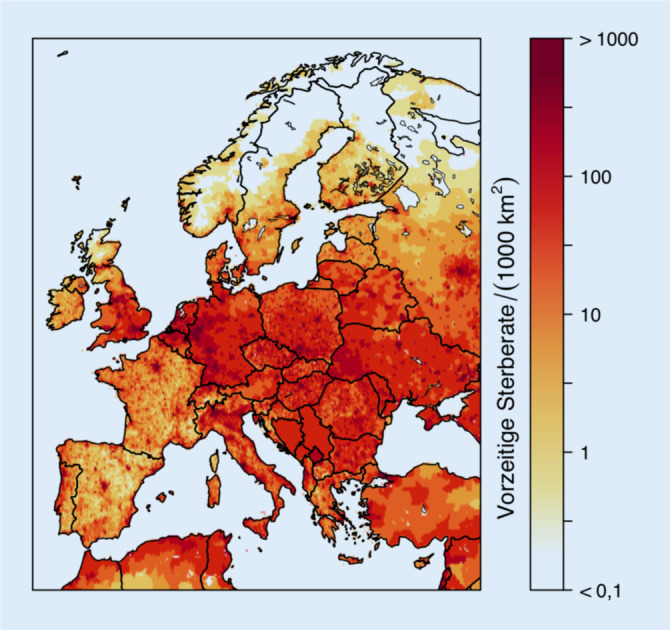

Die damit geschätzte Zahl an zusätzlichen Todesfällen durch die Luftverschmutzung betrug 8,9 Mio. und ist daher mehr als doppelt so hoch wie die von GBD und WHO (4,2 Mio.). Kürzlich berichteten Lelieveld et al. [4], dass auf Basis des GEMM für Europa bis zu 790.000 zusätzliche Todesfälle pro Jahr in Bezug auf PM_2,5_-Exposition und damit mehr als das Doppelte der vorherigen GBD-Schätzung (269.000) resultieren würden (Abb. 2 und 3). Die höchsten Zahlen vorzeitiger Todesfälle ergaben sich interessanterweise hierbei für Deutschland (154 pro 100.000 pro Jahr), gefolgt von Polen (150), Italien (136), Frankreich (105) und dem Vereinigten Königreich (98; [4]). Der Hauptteil der Mortalität war hierbei auf Herz-Kreislauf-Erkrankungen zurückzuführen. Schätzungen von Lelieveld et al. zufolge senkt die Luftverschmutzung durch PM_2,5_ die mittlere Lebenserwartung der Europäer um 2,2 Jahre [4].

**Figure 3:**
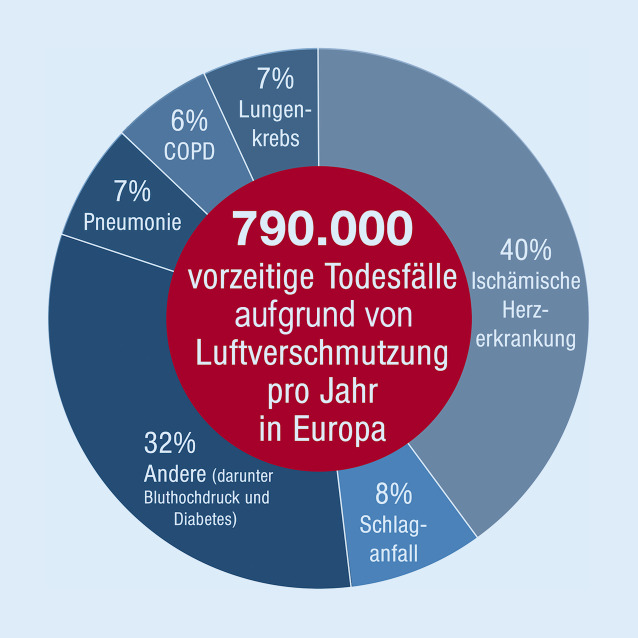

**Figure 4:**
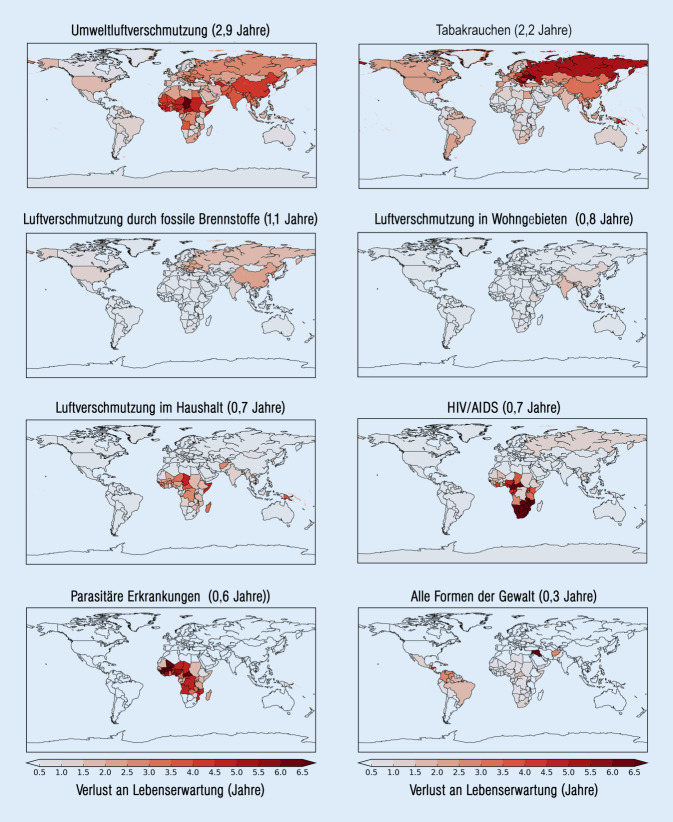

In der neuesten Studie berechneten Lelieveld und Mitarbeiter eine durchschnittliche Verkürzung der weltweiten Pro-Kopf-Lebenserwartung von 2,9 Jahren durch Luftverschmutzung. Im Vergleich dazu wird die Lebenserwartung durch Rauchen um durchschnittlich 2,2 Jahre (7,2 Mio. Todesfälle), durch HIV (humanes Immundefizienzvirus)/AIDS („acquired immunodeficiency syndrome“) um 0,7 Jahre (1 Mio. Todesfälle), durch parasitäre und von Vektoren – also von Lebewesen wie Stechmücken oder Läuse – verursachte Krankheiten wie Malaria um 0,6 Jahre (600.000 Todesfälle) reduziert (Abb. 4; [12]).

## Zusammenhang zwischen Luftverschmutzung und Herz-Kreislauf-Erkrankungen

### Kurzfristige Auswirkungen von Luftverschmutzung

Viele in Metaanalysen eingeschlossene Studien berichten über ein erhöhtes Mortalitätsrisiko in Verbindung mit einer kurzzeitigen Exposition gegenüber Feinstaub, NO_2_ und Ozon. Der durchschnittliche prozentuale Anstieg der Gesamtmortalität für einen Anstieg um 10 µg/m^3^ in der kurzfristigen PM_2,5_-Exposition betrug 1,04 % mit erheblichen Schwankungen zwischen den betrachteten geographischen Regionen [13]. Ebenfalls wurde eine erhöhte Mortalität aufgrund von Atemwegs- und Herz-Kreislauf-Erkrankungen beobachtet [13]. In ostasiatischen Ländern wurden etwas geringere Effektschätzer beobachtet, die gesundheitlichen Auswirkungen sind jedoch aufgrund der sehr hohen Luftverschmutzung deutlich größer [14]. In einer großen Studie, in der Daten aus Europa, aus den USA und aus Kanada analysiert wurden, konnte festgestellt werden, dass ein Anstieg von PM_10_ um 10 µg/m^3^ mit einer Erhöhung der Gesamtmortalität um 0,2–0,6 % assoziiert ist [15].

### Langfristige Auswirkungen von Luftverschmutzung

Langfristige Auswirkungen der Luftverschmutzung auf die vorzeitige Sterblichkeit wurden meist mit PM_2,5_-Konzentrationen in der Umgebungsluft in Verbindung gebracht. Eine Erklärung der American Heart Association (AHA) aus dem Jahr 2010 zeigte im Allgemeinen einen stärkeren Anstieg der Gesamtmortalität im Zusammenhang mit der langfristigen PM_2,5_-Exposition als bei der kurzfristigen Exposition [16]. In einer Überprüfung aus dem Jahr 2013 wurde ein gepoolter Effekt von 6 % für die Gesamt- und von 11 % für die kardiovaskuläre Mortalität bei einem Anstieg von 10 µg/m^3^ PM_2,5_ ermittelt. Daten des ESCAPE(European Study of Cohorts for Air Pollution Effects)-Projekts, bestehend aus 22 Kohortenstudien und mehr als 300.000 Probanden, zeigten eine doppelt so hohe Gesamtmortalität im Vergleich zu früheren Beobachtungen in Bezug auf PM_2,5_ [17]. Diese Assoziationen konnten auch für Personen beobachtet werden, die mit einer mittleren Konzentration von PM_2,5_ von weniger als 15 µg/m^3^ exponiert wurden.

### Kardiovaskuläre Mortalität

Die kurzzeitige PM_2,5_-Exposition erhöht das relative Risiko für einen akuten Herzinfarkt um 2,5 % pro 10 µg/m^3^ [18]. Obwohl diese Risikoerhöhungen relativ gering sind, machen die kurzfristigen Expositionen mit PM_2,5_ weltweit bis zu 5 % der Herzinfarkte aus, da große Massen von Menschen ständig betroffen sind. Die Tatsache, dass die Exposition gegenüber Luftverschmutzungen lebenslang stattfindet, bedeutet wiederum, dass eine kontinuierliche Exposition gegenüber Luftverschmutzung den Prozess der Atherosklerose und rezidivierender kardiovaskulärer Ereignisse fördern kann. In der Tat scheinen Langzeitexpositionen über mehrere Jahre mit einem erhöhten kardiovaskulären Risiko vergesellschaftet zu sein [6, 8, 19].

V. a. können bereits niedrigere Expositionsspiegel in Bezug auf Feinstaub das Risiko für Herz-Kreislauf-Erkrankungen erhöhen. In 2 kanadischen Studien konnten Risikoerhöhungen für ischämische Herzkrankheiten beobachtet werden, obwohl die durchschnittliche PM_2,5_-Konzentration unter 9 µg/m^3^ lag [20]. Ähnliche Ergebnisse wurden in den USA anhand der NIH(National Institutes of Health)-AARP(American Association of Retired Persons)-Kohorten (*n* = 517.043) ermittelt, in denen die Langzeitexposition die kardiovaskuläre Mortalität trotz niedriger PM_2,5_-Konzentrationen zwischen 10 und 13 µg/m^3^ um 10 % pro 10 µg/m^3^ erhöhte [21].

In China gibt es mittlerweile viele Studien mit Betrachtung extrem hoher PM_2,5_-Konzentrationen, die einen Anstieg der akuten kardiovaskulären Mortalität zeigen [22, 23]. In einer Metaanalyse von 59 Studien führte eine Erhöhung der PM_2,5_-Konzentrationen um 10 µg/m^3^ zu einer absoluten Erhöhung der kardiovaskulären Mortalität um 0,63 %, wobei die PM_2,5_-Konzentrationen zwischen 39 und 177 µg/m^3^ lagen [22]. Zudem konnten verschiedene Kohortenstudien mit langen Beobachtungszeiträumen und mit hohen PM_2,5_-Konzentrationen einen erhöhten Einfluss auf die kardiovaskuläre Mortalität bei einer Langzeitexposition nachweisen [24]. Eine wichtige, kürzlich in China durchgeführte Studie hat darüber hinaus gezeigt, dass ein erhöhtes Risiko für Morbidität und Mortalität auch bei sehr hohem Luftverschmutzungsgrad (durchschnittliche PM_2,5_-Konzentrationen von 43,7 µg/m^3^) bestehen bleibt [25]. Dabei konnte ein erhöhtes relatives Risiko für die kardiovaskuläre Mortalität von 9 % pro Anstieg von 10 µg/m^3^ PM_2,5_ beobachtet werden.

### Koronare Ereignisse

Eine systematische Übersicht mit Metaanalyse von Studien zu kurzzeitigen Luftverschmutzungsbelastungen und Herzinfarkt zeigt, dass PM_2,5_ zusammen mit NO_2_ sowie SO_2_ und CO mit einem erhöhten Herzinfarktrisiko verbunden sind [18]. Daten der ESCAPE-Projekts (*n* = 100.166) zeigten einen Anstieg der nichttödlichen akuten koronaren Ereignisse um 13 % bei einer Erhöhung der PM_2,5_-Exposition um 5 µg/m^3^ [17, 26]. Patienten mit bestehender KHK haben ein besonders hohes Risiko. Diesbezüglich konnte gezeigt werden, dass die PM_2,5_-Exposition pro Tag mit einem erhöhten Risiko für ein akutes Koronarsyndrom assoziiert ist. Dies wurde nur bei Personen mit angiographisch nachgewiesener KHK beobachtet, was zu einem Anstieg von ST-Strecken-Hebungs-Myokardinfarkten („ST segment elevation myocardial infarction“, STEMI) führte. Das Langzeitüberleben nach einem akuten Koronarsyndrom wird durch die langfristige PM_2,5_-Exposition ebenfalls reduziert [27].

### Zerebrovaskuläre Erkrankungen

Eine Metaanalyse von 94 Studien bis 2014 ergab, dass eine Erhöhung der PM_2,5_-Konzentration um 10 µg/m^3^ zu einem Anstieg des relativen Risikos für eine Hospitalisierung aufgrund eines Schlaganfalls bzw. Mortalität nach Schlaganfall um 1,1 % führt. Wohnen in der Nähe einer Straße und ein niedriger sozialer Status sind ebenfalls mit dem Schweregrad eines ischämischen Schlaganfalls assoziiert [28]. Ergebnisse der Women’s Health Initiative (WHI) Study zeigten mit die größten Effektschätzer für Schlaganfälle (+35 %) pro 10 µg/m^3^ aufgrund einer zerebrovaskulären Erkrankung nach langfristiger PM_2,5_-Exposition [29].

### Herzinsuffizienz

In einer Metaanalyse von 35 Studien war ein kurzfristiger Anstieg der gasförmigen Bestandteile und PM (sowohl PM_10_ als auch PM_2,5_) mit einem erhöhten Risiko für eine Herzinsuffizienz oder Mortalität durch Herzinsuffizienz verbunden [30]. Ein Anstieg der PM_2,5_-Konzentrationen um 10 µg/m^3^ erhöhte das relative Risiko für Hospitalisierung oder Mortalität um 2,1 % (95 %-Konfidenzintervall [KI]: 1,4–2,8). Eine aktuelle Studie aus China in 26 Städten zeigte, dass ein Anstieg der PM_2,5_-Konzentrationen pro Interquartilsabstand mit einem relativen Anstieg von Hospitalisierung aufgrund einer akuten Herzinsuffizienz um 1,2 % (95 %-KI: 0,5–1,8) verbunden war [31].

### Einfluss auf kardiovaskuläre Risikofaktoren

Es gibt mittlerweile vermehrte Hinweise auf die Entwicklung kardiometabolischer Risikofaktoren wie Bluthochdruck und Insulinresistenz aufgrund von PM_2,5_-Exposition. Der Zusammenhang zwischen Luftverschmutzung und Bluthochdruck wurde in zahlreichen Studien nachgewiesen und war Gegenstand mehrerer aktueller Metaanalysen [32–34]. Ein Anstieg der PM_2,5_-Konzentration um 10 µg/m^3^ war konsistent mit einem Anstieg des systolischen und diastolischen Blutdrucks um 1–3 mm Hg verbunden. Längerfristige Expositionen gegenüber PM_2,5_ waren mit Blutdruckanstiegen und einer erhöhten Prävalenz von Bluthochdruck assoziiert. In methodisch hochwertigen, kontrollierten Studien, in denen Gefäßveränderungen als Konsequenz der Luftverschmutzung bewertet wurden, wurden gleichzeitig Blutdruckänderungen routinemäßig miterfasst [6, 8]. Der Einfluss hinsichtlich Insulinresistenz und Typ-2-Diabetes wurde in früheren Studien überprüft [6, 8]. In einer Metaanalyse von Kohortenstudien stieg das relative Risiko für Diabetes um 39 % pro 10 µg/m^3^ PM_2,5_ [35], und auch in einer kürzlich durchgeführten Metaanalyse von 13 Studien erhöhten PM_2,5_ und NO_2_ das Diabetesrisiko signifikant [36].

## COVID-19 und Luftverschmutzung

Eine kürzlich von Pozzer et al. publizierte Studie berichtet [37], dass die Wahrscheinlichkeit, an einer SARS-CoV-2-Infektion zu sterben, deutlich erhöht ist, wenn man langfristig verschmutzte Luft eingeatmet hat. Hierbei wurde errechnet, dass etwa 15 % der weltweiten Todesfälle durch COVID-19 („coronavirus disease 2019“) auf eine langfristige Exposition gegenüber Luftverschmutzung zurückzuführen sein könnten. Weiterhin ermittelten die Autoren, dass die luftverschmutzungsbedingten COVID-19-Todesfälle in Europa bei 19 %, in Nordamerika bei 17 % und in Ostasien bei 27 % liegen. Die Zahlen sind eine Schätzung des Anteils der COVID-19-Todesfälle, die vermeidbar gewesen wären, wenn die Emissionen aus der Nutzung fossiler Brennstoffe und aus anderen anthropogenen, d. h. vom Menschen verursachten Quellen deutlich niedriger gewesen wären. Für die einzelnen Länder ergeben die Schätzungen der mit Luftverschmutzung in Zusammenhang stehenden COVID-19-Todesfälle ein sehr unterschiedliches Bild: Vergleichsweise hoch ist der Anteil in der Tschechischen Republik mit 29 %, in China mit 27 % und in Deutschland mit 26 % (Abb. 4). Niedriger ist der Anteil beispielsweise in Italien (15 %) oder Brasilien (12 %). Einstellig sind die Werte für Israel (6 %), Australien (3 %) und Neuseeland (1 %). Die Ergebnisse dieser Studie wurden unterstützt durch eine Analyse aus den USA [38], der zufolge pro 1 µg/m^3^ Zunahme des Langzeitdurchschnitts der Feinstaubkonzentration mit einem statistisch signifikanten Anstieg der COVID-19-Mortalität um 8 % zu rechnen ist.

Eine Erklärung dafür wäre, dass die Feinstaubpartikel von der Lunge ins Blut und in die Blutgefäße transmigrieren und dort Entzündungen und starken oxidativen Stress verursachen. Dies wiederum führt zu einer Endotheldysfunktion und zu einer deutlichen Zunahme der Steifheit der Arterien. Auch das Coronavirus gelangt über die Lunge in den Körper und verursacht ähnliche Schäden an den Blutgefäßen, wo auf dem Endothel auch der SARS-CoV-2-Rezeptor ACE(„angiotensin-converting enzyme“)-2 lokalisiert ist. Die Aufnahme des Virus führt zu einem massiven Endothelschaden und zu einer ausgeprägten Entzündungsreaktion, die sich additiv negativ auf das Herz-Kreislauf-System auswirken sowie akute Herzinfarkte, Schlaganfälle bzw. Linksherzdekompensationen bis hin zum akuten Herztod triggern können [39, 40]. Interessanterweise soll Feinstaub auch in der Lage sein, den ACE-2-Rezeptor in der Lunge hochzuregulieren, was die Auswirkungen der SARS-CoV-2-Infektion ebenfalls zusätzlich verstärken würde. Die Ergebnisse legen nahe, dass die Reduzierung der Luftverschmutzung selbst bei relativ niedrigen PM_2,5_-Werten erhebliche Vorteile bringen kann. Man muss verstärkt nach wirksamen Maßnahmen zur Reduktion von anthropogenen Emissionen, die sowohl Luftverschmutzung als auch den Klimawandel verursachen, streben. Der Weg dahin führt über die Minderung von Emissionen. Der Übergang zu einer „grünen“ Wirtschaft mit sauberen, erneuerbaren Energiequellen wird sowohl der Umwelt dienen als auch die öffentliche Gesundheit befördern – lokal durch eine bessere Luftqualität und global durch die Begrenzung des Klimawandels.

## Die Feinstaubgrenzwerte für Europa sind zu hoch

Angesichts des von der WHO empfohlenen Richtwerts von 10 µg/m^3^ für PM_2,5_ kann davon ausgegangen werden, dass weltweit mehr als 91 % der Bevölkerung höheren Konzentrationen ausgesetzt sind (Tab. 1). Die Europäische Union wendet seit 2015 für PM_2,5_ einen mittleren Grenzwert für die Luftqualität von 25 µg/m^3^ an, der 2,5-fach höher ist als der Richtwert der WHO von 10 µg/m^3^. Als Ziel bis 2020 wird ein Wert von 20 µg/m^3^ angegeben, der aufgrund der neuen Datenlage als viel zu hoch angesehen werden muss. Zum Vergleich liegt der Jahresmittelwert in den USA bei 12 µg/m^3^ (seit 2012) und in Kanada bei 10 µg/m^3^ (seit 2015); beide sollen zukünftig weiter reduziert werden (Tab. 1). In Australien liegt der jährliche PM_2,5_-Grenzwert bei 8 µg/m^3^, dieser soll bis 2025 weiter auf 7 µg/m^3^ reduziert werden.

**Table 1:** 

Region	Aktuell PM_2,5_ (µg/m^3^)	Zielwert PM_2,5_ (µg/m^3^)
WHO	10	kA
Europa	25	20 bis 2020^1^
USA	12	kA
Kanada	10	8,8 bis 2020
Australien	8	7 bis 2025

*kA* keine Angabe; *PM*_*2,5*_ „particulate matter“ mit Teilchengröße < 2,5 µm; *WHO* World Health Organization

^1^Der Zielwert 2020 für Europa muss noch durch das Europäische Parlament bestätigt werden

## Persönliche Schutzmaßnahmen gegenüber Feinstaub

Obwohl allgemein anerkannt ist, dass Gesetzgebung, Politik, Regulierung und Technologie für die Durchsetzung einer Reduktion der Luftverschmutzung von entscheidender Bedeutung sind, ist die politische Dynamik, die erforderlich ist, um dies weltweit zu erreichen, derzeit deutlich begrenzt. Persönliche Maßnahmen zur Risikominderung gewinnen daher eine viel größere Bedeutung [41]. Die aktuellen Erfahrungen und Lehren im Zusammenhang mit persönlicher Schutzausrüstung und Risikominderung bei der Reduzierung der SARS-CoV-2-Exposition erinnern stark an diejenigen, die aus ihrer Verwendung zur Bekämpfung der Luftverschmutzung gezogen wurden, obwohl der Schutz je nach Schadstoff unterschiedlich ist [42]. Die Maßnahmen zur Schadensminderung müssen erschwinglich und allgemein anwendbar sein, und das Schutzniveau sollte dem Risiko einer exponierten Bevölkerung entsprechen.

Letzteres würde ein Verständnis des Gesundheitsrisikos des Patienten bzw. der Gemeinschaft und des Expositionsgrades erfordern. Die Notwendigkeit und Dringlichkeit sowie die Intensität einer empfohlenen Intervention müssen mit ihrem potenziellen Nutzen auch gegenüber den Risiken für jeden Einzelnen abgewogen werden (z. B. verschwendete Anstrengungen, Ressourcen, unnötige Bedenken oder mögliche Selbstzufriedenheit des Benutzers). Obwohl bisher für keine Intervention zur Reduzierung der Luftverschmutzung kausal gezeigt werden konnte, dass sie kardiovaskuläre Ereignisse reduziert hat, gibt es einen gesicherten Zusammenhang zwischen erhöhten PM_2,5_-Konzentrationen und kardiovaskulären Ereignissen.

**Figure 5:**
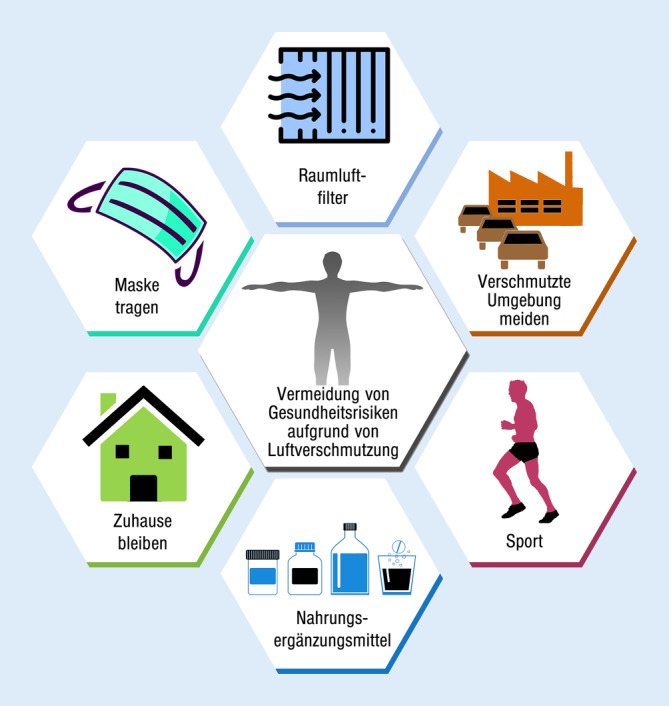

Aktuelle Ansätze zur Minderung der Luftverschmutzung und ihrer Auswirkungen wurden bereits geprüft und lassen sich grob einteilen in ([43]; Abb. 5):

1. aktive Minderung der persönlichen Exposition durch Luftreinigung zu Hause und persönliche Ausrüstung,
2. Änderung des menschlichen Verhaltens zur Reduzierung passiver Expositionen,
3. pharmakologische Ansätze.

Studien mit N95-Atemschutzgeräten unter PM_2,5_-Umgebungsbedingungen bei hohen und niedrigen Expositionsniveaus über einige Stunden haben gezeigt, dass sie den systolischen Blutdruck senken und die Herzfrequenzvariabilitätsindizes (HRV) verbessern [43, 44].

In der einzigen Studie, in der die Minderung von Lärm und Luftverschmutzung untersucht wurde, zeigten sich keine signifikanten Effekte auf den Blutdruck. Die HRV wurden jedoch bei beiden Interventionen verbessert [45]. Gesichtsmasken und Verfahrensmasken (z. B. chirurgische Masken) sind weit verbreitet, filtern jedoch insbesondere dann, wenn sie schlecht sitzen oder während eines langen Zeitraums getragen werden, PM_2,5_ nicht wirksam [46] und können daher nicht zur Verwendung empfohlen werden, wenn N95-Atemschutzgeräte verfügbar sind. Das Schließen von Autofenstern und Klimaanlagen mit Innenraumluftfiltern stellt einen Ansatz dar, der für anfällige Personen von Bedeutung sein könnte, aber nur für diejenigen, die viel Zeit in Transportmikroumgebungen oder zu Hause verbringen. Verhaltensstrategien, wie das Vermeiden von Luftverschmutzung durch Änderung der Reiserouten, Verbleib im Haus bzw. Schließen von Fenstern und Änderung der Aktivität, können dazu beitragen, die Exposition gegenüber Luftverschmutzung zu begrenzen. In einigen Fällen können jedoch unbeabsichtigte Folgen den Nutzen ausgleichen. Ein Beispiel ist das Schließen von Fenstern, um die Exposition im Freien zu begrenzen, mit der dadurch bedingten Gefahr, Luftschadstoffe in Innenräumen zu erhöhen, oder das Begrenzen von Erholung/Bewegung im Freien, um die Exposition in der Umgebung zu verringern. Das letztere Szenario zur Begrenzung der Exposition im Freien wirft einige sehr praktische Fragen hinsichtlich des Risikos des Verlusts des kardiovaskulären Nutzens von Bewegung im Vergleich zum potenziellen Gewinn aufgrund von Vorteilen auf, die durch die Verringerung der Luftverschmutzung entstehen. Modellierungen der Auswirkungen auf die Gesundheit und epidemiologische Studien haben gezeigt, dass die Vorteile von Aerobic-Übungen fast immer das Risiko einer Luftverschmutzung über einen Konzentrationsbereich und für lange Trainingsdauern bei normalen Personen (>75 min) übersteigen.

Nach aktuellen Erkenntnissen kann eine Empfehlung zur Vermeidung von Aktivitäten im Freien in Gebieten mit hoher PM_2,5_-Belastung angesichts des geringen absoluten Risikos für kardiovaskuläre oder respiratorische Ereignisse nicht ausgesprochen werden. Auf der anderen Seite ist es eine vernünftige Maßnahme, Patienten mit vorab festgestellten Herz-Kreislauf-Erkrankungen zu raten, weiterhin mehr als 400 m von Hauptstraßen entfernt zu bleiben, um eine Exposition gegenüber Verkehrsschadstoffen zu vermeiden, obwohl die Datenlage zu diesem Punkt alles andere als eindeutig ist [47].

Rezeptfreie Medikamente, wie z. B. Vitamine, sind in der Regel wirkungslos und können derzeit keinen Schutz vor durch Luftverschmutzung verursachten gesundheitlichen Auswirkungen bieten. Die Verwendung von Medikamenten zur primären und sekundären Prävention von KHK sollte jedoch gefördert werden, wenn dies aus zusätzlichen Gründen indiziert ist [47].

## Fazit für die Praxis

- Bedingt durch die Luftverschmutzung (PM_2,5_ und Ozon) gibt es weltweit 8,9 Mio. vorzeitige Todesfälle pro Jahr.
- Für Europa, inklusive der Türkei, der Ukraine und West-Russland, werden ca. 800.000 vorzeitige Todesfälle pro Jahr berechnet, wobei hier insbesondere koronare Herzkrankheit und Schlaganfall mit etwa 50 % vorzeitigen Todesfällen dominieren.
- Die globale Reduktion der Lebenserwartung durch Luftverschmutzung, v. a. durch Feinstaub, liegt bei geschätzten 2,9 Jahren pro Jahr. Dies bedeutet in der Summe für die öffentliche Gesundheit, dass Feinstaub eine ähnliche Bedeutung wie das Rauchen hat.
- Der für Europa geltende Grenzwert von 25 µg/m^3^ muss drastisch reduziert werden.
- Die Luftverschmutzung verschlechtert die Prognose von Patienten, die an COVID-19 erkranken.
- Es gibt jedoch keinen Impfstoff gegen schlechte Luftqualität und den Klimawandel.
- Zudem muss Luftverschmutzung als kardiovaskulärer Risikofaktor anerkannt und in den Leitlinien für Prävention, Herzinfarkt und Schlaganfall der ESC bzw. der AHA und des ACC erwähnt und entsprechend bewertet werden.
